# Loneliness as a sex-specific risk factor for cognitive aging

**DOI:** 10.3389/fnhum.2026.1784613

**Published:** 2026-02-24

**Authors:** Alison Warren

**Affiliations:** 1 Department of Clinical Research and Leadership, George Washington University School of Medicine and Health Sciences, Washington, DC, United States; 2 Frame-Corr Laboratory, Department of Clinical Research and Leadership, George Washington University School of Medicine and Health Sciences, Washington, DC, United States; 3 Department of Continuing Education, Harvard University Extension School, Cambridge, MA, United States

**Keywords:** Alzheimer’s disease, dementia, cognitive aging, loneliness, social isolation, sex difference, gender difference

## Abstract

Loneliness has emerged as a robust risk factor for adverse cognitive outcomes, including accelerated cognitive decline and increased dementia risk. However, accumulating evidence suggests that the cognitive consequences of loneliness may be sex-specific, with women experiencing disproportionate exposure, vulnerability, and downstream effects across the life course. This mini-review synthesizes epidemiological, psychosocial, and neurobiological evidence linking loneliness to cognitive aging in women, highlighting sex differences in prevalence, social role exposures (e.g., caregiving, widowhood), stress responsivity, and neuroendocrine and inflammatory pathways. Findings from longitudinal cohort studies are reviewed, demonstrating stronger associations between loneliness and cognitive decline in women, alongside emerging mechanistic research implicating hypothalamic–pituitary–adrenal (HPA) axis dysregulation, immune activation, and hippocampal vulnerability. Critical gaps in aging neuroscience, including limited sex-disaggregated analyses and undermeasurement of relational and social variables, require urgent attention. A sex-sensitive framework for integrating loneliness into cognitive aging research and prevention strategies, emphasizing targeted assessment and intervention approaches that reflect women’s lived social contexts, holds promise for advancing precision prevention in women’s brain health. Framing loneliness as a modifiable, sex-specific risk factor offers a novel avenue for precision prevention in women’s cognitive aging.

## 1 Introduction

Over two-thirds of the population will experience loneliness at some point in their lives (Blodgett et al., 2025). Defined as the subjective distress arising from a perceived gap between desired and actual social relationships, loneliness has emerged as a critical determinant of health and aging (Hawkley and Cacioppo, 2010). To further expound, emotional loneliness refers to the absence of close, intimate attachment figures, whereas social loneliness reflects a lack of broader social integration or group belonging; these subtypes exhibit distinct psychosocial and neurocognitive correlates across the lifespan (Tao et al., 2022; Manoli et al., 2022). Although long viewed primarily as a social or psychological issue, accumulating evidence positions loneliness as a biopsychosocial risk factor that contributes to morbidity and mortality comparable to traditional cardiovascular risks such as smoking or obesity (Holt-Lunstad et al., 2015; Warren, 2025). Its impact is particularly salient in later life, when shrinking social networks, bereavement, and declining mobility heighten vulnerability to both social isolation and the subjective experience of disconnection (Courtin and Knapp, 2017). Notably, the 2024 *Lancet* Commission on Dementia Prevention identifies loneliness and social isolation as one of the 14 key modifiable risk factors for dementia and highlights social engagement and loneliness reduction as among the most impactful strategies for dementia prevention, underscoring the public health importance of early identification and intervention (Livingston et al., 2024).

Recent large-scale cohort and meta-analytic studies indicate that loneliness and social isolation are not interchangeable constructs: isolation reflects the objective absence of contact, whereas loneliness reflects subjective dissatisfaction with one’s relationships (Coyle and Dugan, 2012; Leigh-Hunt et al., 2017). Both phenomena are independently associated with accelerated cognitive decline, dementia, and increased mortality (Ren et al., 2023; Salinas et al., 2022; Shen et al., 2022). However, mounting evidence suggests that these effects are not uniform across sexes. Women tend to report higher levels of perceived loneliness, whereas men experience greater objective social isolation (Maes et al., 2019; Umberson et al., 2022). These sex differences have distinct health consequences: a recent study found that social isolation independently predicts mortality in both men and women, especially from cardiovascular-related causes, whereas loneliness significantly increases overall mortality risk in women but not in men (Vallée, 2025). The health consequences of loneliness therefore may be biologically and socially sex-differentiated—driven by hormonal regulation of the hypothalamic–pituitary–adrenal (HPA) axis, gendered patterns of coping, and differences in help-seeking and social network composition (Díaz-Mardomingo et al., 2023; Qi et al., 2023).

From a neurobiological perspective, chronic loneliness activates stress-response pathways that promote glucocorticoid dysregulation, inflammation, and structural brain changes, particularly within the hippocampus and prefrontal cortex, which are regions critical to memory and executive function (Tao et al., 2022; Díaz-Mardomingo et al., 2023; Cacioppo et al., 2015). Persistent social disconnection has been linked to elevated inflammatory markers (e.g., C-reactive protein, interleukin-6) altered cortisol regulation, and diminished brain volumes and enlarged ventricular volume, all biomarkers associated with cognitive aging (Díaz-Mardomingo et al., 2023; Qi et al., 2023).

Loneliness has been linked to subjective cognitive decline, cardiovascular disease, and depressive symptoms, but the intertwined nature of these neuropsychiatric factors highlight the need for further research to disentangle their distinct contributions to biomarkers of brain pathology (Zapater-Fajarí et al., 2025). Social isolation and loneliness exhibit distinct relationships with depressive symptoms, with depression potentially preceding social isolation and showing a bidirectional association with loneliness, which may function as both an antecedent and a consequence (Luo, 2023); although loneliness and depression frequently co-occur (Luo, 2023; Akinyemi et al., 2025), they remain distinct constructs and are each recognized among the 14 modifiable risk factors for dementia., Importantly, several longitudinal studies, including analyses of the Framingham Heart Study and UK Biobank, suggest that loneliness is associated with dementia onset, cognitive decline, and mortality; however, the extent to which these associations are independent of depression symptomology varies across studies and outcomes (Shen et al., 2022; Vallée, 2025; Zapater-Fajarí et al., 2025; Akhter-Khan et al., 2021). While some investigations report effects that persist after adjustment for depression, others indicate partial or full mediation, particularly for white matter abnormalities and subjective cognitive decline (Zapater-Fajarí et al., 2025), highlighting the need for cautious interpretation and age-specific modeling.

Despite this expanding body of evidence, the role of sex in moderating these associations remains underrecognized and under-characterized. Women’s greater emotional expressivity and social proclivity may amplify the perceived burden of loneliness, while men’s tendency toward emotional stoicism and reliance on spousal support may mask loneliness but intensify its physiological toll when social bonds are lost (Umberson et al., 2022; Ratcliffe et al., 2024). This growing recognition has prompted calls for sex-sensitive models of social health and cognitive aging, including frameworks that integrate biological, psychological, and sociocultural dimensions of loneliness (Guarnera et al., 2023; Oken et al., 2024).

This review synthesizes recent empirical and mechanistic evidence to elucidate how loneliness operates as a sex-specific risk factor for cognitive decline. By integrating epidemiological, neurobiological, and psychosocial perspectives, it aims to clarify both the shared and distinct pathways through which loneliness contributes to cognitive aging in men and women, and to identify implications for sex-informed prevention and intervention strategies.

## 2 Epidemiology of loneliness and cognitive aging

### 2.1 Sex differences in loneliness across the lifespan

Loneliness is a widespread psychosocial phenomenon with sex-based differences in prevalence, trajectory, and impact still under exploration. Longitudinal studies report that women consistently experience higher levels of loneliness than men throughout the lifespan (Nicolaisen and Thorsen, 2024). However, variables across the sexes and life stages are worth noting, as the differences in the experience of loneliness may be a more important consideration than the prevalence alone. According to a meta-analysis by Maes et al. (2019), which examined 751 effect sizes (*N* = 399, 798) and found that sex differences in loneliness across the lifespan are minimal, with only small, context-dependent variations, challenging the common assumption that loneliness can be conceptualized as a binary construct between sexes. Life stage may tip the scale in this regard, as seen in Nicolaisen and Thorsen (2024) 15-year longitudinal study in Norway to examine sex differences in loneliness among adults aged 40 and older. They found that while men reported higher initial loneliness levels, women experienced a steeper increase in loneliness over time, particularly after age 70. Further, adolescent loneliness has become an increasingly critical problem in both sexes (Kirwan et al., 2025; Twenge et al., 2021). A recent study by Liu et al. (2026) found that loneliness was more prevalent among females than males overall, though prevalence increased with age among males and exceeded that of females after mid-adolescence. While depression, anxiety, and poor sleep quality were associated with loneliness in both sexes, non-suicidal self-injury and poor dietary habits (e.g., low vegetable intake, binge eating) were independent correlates only in females, whereas age and heavy academic pressure were uniquely associated with loneliness in males (Liu et al., 2026).

These findings highlight that sex differences in loneliness are dynamic rather than static, suggesting the need for age- and sex-sensitive interventions. Building on this, recent scholarship has challenged traditional frameworks that treat loneliness as a uniform or binary experience across genders. Rather than solely focusing on prevalence, researchers argue that loneliness is a socially constructed phenomenon, deeply shaped by gender norms, roles, and relational expectations (Barreto et al., 2025). This perspective emphasizes that men and women may express, experience, and cope with loneliness in qualitatively different ways—differences that are often overlooked by conventional, quantitative methodologies. As such, more intersectional and context-sensitive approaches are needed to capture the nuanced and sex-based realities of loneliness across diverse life stages and social conditions. Thus, contextual factors, such as loneliness type, life stage, age-normative expectations, and other social determinants of health factor sex differences observed in the experience of loneliness (Maes et al., 2019).

Gender differences are also reflected in patterns of social connectedness. Women tend to maintain broader, more emotionally intimate social networks, while men are more reliant on spouses or singular relationships. Umberson et al. (2022) emphasize that older men are structurally more isolated but often less likely to report feeling lonely due to gendered norms around emotional expression. Further supporting sex-differentiated psychosocial pathways, a population-based study of adults aged 55–75 found that higher loneliness was associated with poorer psychological health outcomes in men, but not women, suggesting that emotional distress may manifest differently across sexes despite similar loneliness exposure (Crespo-Sanmiguel et al., 2022). These findings align with life-course sociological models indicating that men’s greater reliance on spousal ties may confer vulnerability to psychological distress when those bonds are disrupted, even when loneliness is under-reported due to gendered norms surrounding emotional expression (Umberson et al., 2022).

Cross-national data supports this trend. In a longitudinal analysis from the English Longitudinal Study of Ageing (ELSA), Ratcliffe et al. (2024) found that older men under-report loneliness despite similar objective risk profiles. Their coping strategies, such as increased alcohol consumption, also differed sharply by sex. Meanwhile, in Myanmar, Htun et al. (2025) showed that older women were more emotionally affected by pandemic-related isolation, reporting significant declines in perceived social support and increased loneliness during COVID-19 lockdowns.

### 2.2 Sex differences in cognitive aging and dementia risk

The lifetime risk of Alzheimer’s disease and related dementias (ADRD) is markedly higher in women, who account for two-thirds of all ADRD cases globally (Alzheimer’s Association, 2025). While this has historically been attributed to greater female longevity, mounting evidence suggests psychosocial risk exposures—including loneliness—may contribute to this disparity.

Loneliness is a well-established risk factor for dementia in both sexes (Livingston et al., 2024; Salinas et al., 2022). A landmark study by Salinas et al. (2022) using data from the Framingham Heart Study found that loneliness was associated with a 54% higher risk of dementia over a 10-year period, independent of *APOE-ε4* status, depression, and cardiovascular risk. While much prior research has shown that loneliness is linked to cognitive decline, most studies have not differentiated between types or chronicity of loneliness. To explore this gap, Tao et al. (2022), using longitudinal data from the Framingham Heart Study, found that persistent loneliness, as opposed to transient or occasional loneliness, was strongly associated with cognitive decline and structural brain changes, particularly among women and individuals with the *APOE4* genetic risk for Alzheimer’s disease. Persistent loneliness was linked to smaller volumes in key brain regions such as the frontal and temporal lobes, hippocampus, and enlarged ventricles, which are early markers of Alzheimer’s pathology (Tao et al., 2022). These findings underscore the importance of identifying and addressing chronic loneliness in midlife, especially in high-risk groups, to help prevent neurodegeneration and cognitive impairment with aging.

In contrast, Shen et al. (2022), using UK Biobank data (*N* = 462,619), found that social isolation, but not loneliness, was independently predictive of dementia, while the effect of loneliness was fully mediated by depression. These associations were consistent in men and women (Shen et al., 2022). A study involving community-dwelling older adults by Joyce et al. (2022; *N* = 11, 498) social isolation and low social support were associated with poorer baseline cognitive function in women but not men, while loneliness showed no consistent cross-sectional association with cognition. Social health, reflected in one’s level of content with social interactions, engagement, support, and relationships, did not predict cognitive decline or incident dementia over follow-up, although limited power for sex-stratified analyses suggests potential gender-dependent effects with longer observation (Joyce et al., 2022).

Lastly, a study by Akhter-Khan et al. (2021) showed that persistent loneliness was linked to nearly double the risk of developing Alzheimer’s disease over an 18-year period. Although not sex-stratified, participants experiencing transient, incident, or persistent loneliness—compared to those who did not report loneliness—were more likely to be women, live independently, be widowed, experience depression, and have fewer social connections (Akhter-Khan et al., 2021).

Collectively, these findings underscore the sex-based epidemiology of loneliness and cognitive aging. While men may be more socially isolated structurally, women appear more emotionally vulnerable to loneliness, and this vulnerability may exert disproportionate neurocognitive consequences. Given the interaction between sex, loneliness type, and dementia risk, future research and intervention efforts must prioritize sex-disaggregated analysis and targeted prevention models.

## 3 Psychosocial pathways linking loneliness and cognition in women

### 3.1 Psychosocial stress and cognitive burden

Loneliness is more than a transient emotional experience, it is a chronic psychosocial stressor with measurable effects on health and cognition. Prolonged loneliness activates the brain’s threat surveillance systems, triggering behavioral hypervigilance and increased salience of social threats, as pioneered in neuroimaging studies by Cacioppo et al. (2009) and later supported by several studies (Tao et al., 2022; Du et al., 2022; Spithoven et al., 2017; Wilkialis et al., 2021). This chronic vigilance state leads to executive overload and attentional resource depletion, key early markers of cognitive decline.

In the context of sex, women may be particularly affected due to the internalization of emotional distress, a phenomenon deeply tied to socialized caregiving roles and relational expectations (Kaiser et al., 2025). Women’s stronger emotional burden associated with the loneliness experience, as reflected in higher emotional loneliness scores, may further heighten cognitive load, depression, and anxiety (Lazuras et al., 2024). This aligns with recent findings revealing that loneliness was significantly correlated with cognitive deficits (particularly in memory and attention) among women with schizophrenia, but not in men (Chi et al., 2024). Although schizophrenia is etiologically distinct from Alzheimer’s disease, these findings are informative for aging research because they illustrate sex-specific cognitive vulnerability to loneliness under conditions of chronic social stress and neuroinflammatory burden, which reflect mechanisms that are also implicated in normative cognitive aging (Lam et al., 2021).

Importantly, Umberson et al. (2022) emphasize that loneliness is deeply gendered and life-stage dependent. Life-stage differences are critical: loneliness in early adulthood is often episodic and peer-related, whereas in later life it is more frequently driven by bereavement, caregiving strain, and health-related role loss, which are factors that disproportionately affect women and may exert cumulative neurocognitive effects (Umberson et al., 2022; Nicolaisen and Thorsen, 2024). In this regard, Umberson et al. (2022) found that men are more likely to experience structural social isolation due to smaller, spouse-centered networks, while women report greater subjective loneliness despite broader social ties. These gendered patterns, shaped by social norms and life course transitions, lead to different health outcomes (Umberson et al., 2022). The differences in experiential emotional processing may contribute to the heightened psychological and cognitive vulnerability to loneliness observed in women.

### 3.2 Biological mechanisms: HPA axis and neuroinflammation

The link between loneliness and cognitive aging is mediated in part by well-characterized biological stress pathways: chronic loneliness is associated with activation of stress-response systems, including hypothalamic–pituitary–adrenal (HPA) axis dysregulation and altered cortisol patterns, which may contribute to downstream physiological wear (Warren, 2025), heightened amygdala reactivity, poorer autonomic regulation, and cognitive vulnerability (Hawkley and Cacioppo, 2010; Díaz-Mardomingo et al., 2023; Cacioppo et al., 2015). Persistently cortisol dysregulation has been linked to smaller hippocampal volume, impaired hippocampal function, and reduced neurogenesis, supporting a plausible pathway from chronic stress exposure to brain changes associated with cognitive decline and Alzheimer’s disease risk (Dronse et al., 2023). In parallel, loneliness is associated with immune dysregulation, including pro-inflammatory transcriptional signaling and elevated circulating markers such as interleukin-6 and C-reactive protein (Hawkley and Cacioppo, 2010; Cacioppo et al., 2015; Hawkley et al., 2007). Importantly, these inflammatory markers are best interpreted as indicators of neurobiological vulnerability rather than direct evidence of classical Alzheimer’s disease pathology (amyloid-*β* or tau accumulation); mechanistically, sustained peripheral inflammation may contribute to neuroinflammation and subsequent neurodegeneration through microglial priming, astrocytic activation, and impaired neurovascular integrity (Heneka et al., 2025). Consistent with an inflammation-related vulnerability model, large cohort neuroimaging studies show that loneliness is associated with smaller total cerebral volume and greater white matter injury (Salinas et al., 2022), and UK Biobank findings similarly link social disconnection-related risk patterns to later dementia with neurostructural correlates such as white matter changes and cognitive differences (Shen et al., 2022), supporting the cautious interpretation that loneliness may contribute to cognitive aging through stress–immune pathways that preferentially impact hippocampal and white matter integrity even when direct measures of amyloid and tau are not available.

### 3.3 Sex differences in biological response

Emerging evidence suggests that men and women may exhibit distinct neurobiological responses to loneliness and its associated stress response. Animal models and human neuroendocrine studies indicate that females may be more reactive to social stress at both neural and immune levels (Handa et al., 2022), and may be more susceptible to the depressive consequences of stress (Bekhbat and Neigh, 2018). Compared to men, women are more susceptible to mood and behavioral changes triggered by stress-induced inflammation, such as heightened feelings of social disconnection and loneliness, even during brief periods of inflammation, which, in turn, raises their vulnerability to depression (Mengelkoch and Slavich, 2024). This aligns with findings from Tao et al. (2022), in which persistent loneliness was associated with reduced volume in the temporal lobe, hippocampus, and frontal lobe, along with larger lateral ventricles across all loneliness types in women. These structural brain changes were not observed in men, except for a link between incident loneliness and increased hippocampal volume, which remained even after accounting for other influencing factors (Tao et al., 2022).

Moreover, the neuroinflammatory consequences of loneliness appear more pronounced in postmenopausal women, possibly due to the loss of estrogen’s neuroprotective and anti-inflammatory effects (Handa et al., 2022; Bekhbat and Neigh, 2018; Mengelkoch and Slavich, 2024; Bustamante-Barrientos et al., 2021). This might partially explain why loneliness more strongly predicts Alzheimer’s disease risk in women (Akhter-Khan et al., 2021).

Together, psychosocial and biological evidence underscores that loneliness contributes to cognitive aging through both behavioral and physiological mechanisms. While social isolation impacts both sexes, women show greater cognitive vulnerability e to the emotional and neuroinflammatory burdens of loneliness. Understanding these sex-based divergences is essential for developing precision health interventions targeting cognitive decline in aging populations.

## 4 Neurocognitive correlates of loneliness

### 4.1 Structural brain changes

Loneliness has been associated with distinct neuroanatomical and functional brain changes, many of which overlap with biomarkers of cognitive impairment and Alzheimer’s disease (Zapater-Fajarí et al., 2025; Lam et al., 2021). In the aforementioned study by Tao et al. (2022) using data from the Framingham Heart Study, emotionally lonely individuals exhibited significantly lower cortical thickness in the prefrontal, temporal, and parietal lobes—regions heavily implicated in aging-related cognitive decline. Critically, the authors observed sex-specific effects, with women showing stronger associations between emotional loneliness and reduced cortical thickness, particularly in the dorsolateral prefrontal cortex and hippocampus, which are crucial for working memory and executive control.

Similarly, Salinas et al. (2022) reported that loneliness was linked to smaller total cerebral volume and greater white matter injury after adjustment for common Alzheimer’s disease risk factors, including *APOE-ε4* genotype and depressive symptoms. These findings, drawn from the same Framingham cohort, align with known early biomarkers of Alzheimer’s disease and suggest loneliness as a preclinical risk factor. Notably, associations were most pronounced in adults under 80 years of age without elevated genetic risk, indicating that loneliness may accelerate brain aging even in otherwise low-risk individuals. Further, in a cohort of 215 cognitively unimpaired 70-year-old adults with cerebrospinal fluid and neuroimaging biomarkers, Zapater-Fajarí et al. (2025) found that loneliness was most strongly associated with depressive symptomatology and cerebrovascular disease and contributed to distinguishing individuals with subjective cognitive decline. However, when depressive symptoms and Alzheimer’s disease biomarkers were accounted for, loneliness was no longer an independent predictor of SCD, highlighting the complex and overlapping relationships among loneliness, depression, and brain pathology (Zapater-Fajarí et al., 2025).

### 4.2 Functional brain connectivity and social cognition networks

Loneliness also disrupts functional connectivity in major neural networks. The default mode network (DMN)—involved in self-referential thought, memory, and future planning—is often dysregulated in lonely individuals (Lam et al., 2021; Spreng et al., 2020). Further, loneliness is associated with altered DMN activity, particularly between the posterior cingulate cortex, medial prefrontal cortex, and hippocampus, all of which are critical for episodic memory and are early targets of Alzheimer’s pathology and other forms of cerebrovascular disease (Lam et al., 2021; Lao et al., 2024).

Additionally, functional MRI studies have shown that lonely individuals exhibit heightened amygdala activation (Spreng et al., 2020; Lieberz et al., 2021) and altered salience network engagement (Vitale and Smith, 2022), reflecting a shift toward threat-related social processing. These patterns may promote chronic stress reactivity, which create downstream effects that contribute to hippocampal damage and impaired cognitive performance (Hawkley and Cacioppo, 2010; Cacioppo et al., 2015; Lam et al., 2021).

### 4.3 Sex differences in neural susceptibility

Accumulating evidence suggests that female brains may be more susceptible to loneliness-related structural and functional changes. In the Tao et al. (2022) study, emotionally lonely women showed greater deficits in executive function and working memory, paralleling more pronounced reductions in cortical thickness compared to men.

These differences may be linked to hormonal modulation. The decline in estrogen during menopause reduces neuroprotection, impacts cerebral perfusion, and increases inflammation—factors that may amplify the neurobiological impact of loneliness in women. Moreover, women tend to engage emotion-centric brain networks more heavily, which may make them more vulnerable to disconnection when relational needs are unmet.

Complementary behavioral findings from Chi et al. (2024) show that lonely female patients with schizophrenia had significant deficits in attention, language, and delayed memory, while no such associations were observed in men. These neurocognitive deficits mirror known patterns of female-prevalent Alzheimer’s progression and further support a sex-specific cognitive impact of loneliness.

Loneliness leaves a measurable imprint on the aging brain, with associations spanning cortical atrophy, white matter degradation, altered functional connectivity, and diminished cognitive performance. Importantly, neurological susceptibility to the inflammatory effects of loneliness appears to be more pronounced in women, potentially due to hormonal, behavioral, and social factors that increase risk for neuropsychological disorders (Tao et al., 2022; Mengelkoch and Slavich, 2024; Ali et al., 2023). These findings suggest that loneliness may be a neurobiological amplifier of cognitive aging in women, highlighting the need for targeted interventions and further sex-stratified longitudinal research.

## 5 Loneliness as an early marker and modifiable risk factor

There is growing consensus in the literature that loneliness not only correlates with cognitive decline but may serve as a sentinel marker of preclinical neurocognitive vulnerability. Importantly, its modifiable nature distinguishes it from other non-behavioral risk factors (e.g., *APOE* genotype), rendering it an actionable target for early intervention and dementia prevention.

### 5.1 Loneliness as a preclinical Indicator

Longitudinal studies have established that loneliness often precedes detectable cognitive impairment. In the Framingham Heart Study, Salinas et al. (2022) demonstrated that loneliness predicted increased dementia risk 10 years prior to clinical onset, even after controlling for *APOE-ε4* status and depression. Notably, Tao et al. (2022) found that emotionally lonely individuals already exhibited brain structure changes and reduced cognitive performance, suggesting that loneliness may reflect underlying neuropathological changes before standard cognitive screenings can detect them. Moreover, growing evidence indicates that loneliness is linked to polypharmacy and the use of high-risk medications in later life, with severe loneliness independently predicting polypharmacy among older women but not men (Im et al., 2023). These findings suggest that clinicians should incorporate loneliness into medication reviews and deprescribing strategies to reduce medication-related harms, particularly for older female patients (Im et al., 2023).

Other large-scale studies corroborate this timeline. For instance, Shen et al. (2022) analyzed UK Biobank data (N > 460,000) and found that social isolation (not loneliness) independently predicted dementia, while loneliness’s effects were mediated by depression—underscoring the importance of differentiating between emotional and structural disconnection in early screening.

### 5.2 Depression, loneliness, and cognitive risk

The prevalence of loneliness may vary between genders across the lifespan, but depression is consistently twice as likely in women and more incapacitating (Aneja and Kaur, 2025; Liu et al., 2020). Loneliness and depression are closely intertwined yet distinct constructs that are statistically separable (Salinas et al., 2022), with bidirectional relationships that complicate causal inference in cognitive aging research. While loneliness may precipitate depressive symptoms via chronic stress and heightened social threat vigilance, depression can also intensify perceived social disconnection through anhedonia, withdrawal, and negative cognitive bias (Hawkley and Cacioppo, 2010; Cacioppo et al., 2015; Slavich and Irwin, 2014; Finley and Schaefer, 2022). Several large cohort studies demonstrate that loneliness predicts dementia risk even after adjusting for depressive symptoms (Salinas et al., 2022; Akhter-Khan et al., 2021), whereas others report partial or full mediation, particularly for white matter changes and subjective cognitive decline (Shen et al., 2022; Zapater-Fajarí et al., 2025). These mixed findings suggest that depression may function both as a mediator and a modifier of loneliness-related cognitive risk, with effects varying by age, sex, and outcome. Disentangling these pathways remains a critical priority for future longitudinal and mechanistic studies.

### 5.3 Modifiability and intervention potential

Loneliness is widely recognized as a modifiable risk factor for cognitive decline (Livingston et al., 2024) and other long-term health conditions (Hounkpatin et al., 2024). Systematic reviews and WHO guidelines emphasize that community engagement, structured social interventions, and targeted psychotherapy can reduce loneliness and potentially slow cognitive deterioration (WHO Commission on Social Connection, 2025). As reinforced by the Lancet Commission, lifestyle factors are potent modifiable risk factors that can significantly reduce the risk of developing dementia dementia (Livingston et al., 2024). Further support is offered in the US POINTER randomized clinical trial, which demonstrated that a structured, higher-intensity multidomain lifestyle intervention (e.g., regular physical activity, adherence to the MIND diet, cognitive stimulation, and social engagement) produced significantly greater improvements in global cognitive function over 2 years compared with a self-guided intervention in older adults at elevated risk for dementia (Baker et al., 2025). Although the trial was not designed to examine sex-specific effects, these findings support social engagement as a key modifiable component of dementia prevention and provide empirical justification for targeting loneliness reduction within multidomain risk-reduction strategies (Salinas et al., 2022).

Intervention trials in aging populations show that cognitive training combined with social participation improves executive function and reduces subjective loneliness (Akhter-Khan et al., 2021). Moreover, digital solutions like telehealth, virtual companionship, and social media have shown promise in reducing the perception of loneliness (Kusumota et al., 2022), especially for older persons living alone who represent a demographic highly vulnerable to chronic loneliness (Blodgett et al., 2025; Hoang et al., 2022).

Importantly, sex-specific tailoring enhances intervention effectiveness. Women tend to respond more positively to emotionally supportive programs, while men may benefit more from task-oriented or activity-based interventions, according to data from the English Longitudinal Study of Ageing (Ratcliffe et al., 2024). Interventions that fail to account for these differences may unintentionally widen disparities in cognitive aging outcomes.

### 5.4 Policy and public health implications

Given loneliness’s demonstrated role in accelerating cognitive decline, and its modifiability, it qualifies as a prime candidate for inclusion in public health dementia risk reduction frameworks. The 2020 Lancet Commission on Dementia Prevention recognized social engagement as a protective factor, and recent expansions advocate for loneliness screening as part of annual cognitive wellness assessments in primary care (Livingston et al., 2024; Mullen et al., 2019; Pollak et al., 2025).

Moreover, programs targeting loneliness should integrate sex-sensitive outreach, especially for older women, widows, and caregivers—populations disproportionately affected by social disconnection during life crises as further highlighted by the COVID-19 pandemic (Bhat et al., 2024). As evidence accumulates, incorporating loneliness as a clinical biomarker and psychosocial target may transform how dementia risk is conceptualized and managed.

Loneliness is both an early indicator of cognitive vulnerability and a modifiable behavioral risk factor, positioning it at the intersection of prevention and detection. Robust sex-specific data further suggests that loneliness plays a more consequential role in women’s cognitive aging. Future clinical models that incorporate loneliness into dementia risk profiles—alongside neuroimaging, genetic, and metabolic data—will likely offer the most holistic and equitable strategy for brain health promotion in aging populations.

## 6 Gaps and limitations in aging neuroscience

While research has made significant strides in identifying loneliness as a risk factor for cognitive decline, multiple critical limitations remain. Chief among them are the underutilization of sex-disaggregated models, the oversimplification of social-relational variables, and a pervasive lack of intersectional frameworks that consider how gender interacts with other dimensions of identity (Barreto et al., 2021; Gustafsson et al., 2022; Li and Spini, 2022). These limitations restrict both scientific understanding and the development of equitable, targeted interventions.

### 6.1 Underrepresentation of sex-disaggregated analyses

Despite a growing body of evidence suggesting that loneliness affects women and men differently, many large-scale studies continue to treat sex as a statistical control rather than an analytical lens. In landmark longitudinal studies such as UK Biobank (Shen et al., 2022) and Framingham (Salinas et al., 2022), findings are pooled across sexes, missing potentially meaningful interaction effects between sex and loneliness on cognitive outcomes.

While studies like Tao et al. (2022) provide sex-stratified analyses showing greater cortical thinning in emotionally lonely women, such designs are still the exception. Furthermore, few studies employ formal moderation or mediation models to test how sex might condition the relationship between loneliness and brain aging.

Additionally, loneliness is often treated as a secondary covariate in models of cognitive decline, rather than a primary psychosocial exposure. This de-prioritization reduces visibility into its mechanistic roles and reinforces the medicalization of cognitive aging as a purely neurobiological process.

### 6.2 Undermeasurement of relational and social variables

A second major limitation is the reductionist measurement of loneliness and social connection. Most studies rely on brief, unidimensional scales (e.g., single-item measures or the short UCLA scale), which excel at rapid screening (Hughes et al., 2004; Russell et al., 1980), but could obscure the qualitative nuances of relational life.

Variables in need of improvement:

- Relationship quality, including emotional reciprocity or conflict
- Caregiving burden, which disproportionately affects older women and widows
- Social role context, such as living alone, widowhood, and retirement

These omissions are consequential. For example, caregiving strain is a well-documented stressor linked to both loneliness and cognitive decline, yet is almost never included in neurocognitive models. Similarly, emotional vs. social loneliness, which may vary across the lifespan (Manoli et al., 2022), remains underutilized in many cohort datasets.

Without deeper, more contextualized measurement, studies risk oversimplifying the lived experience of loneliness, and thereby misrepresenting its contribution to cognitive risk.

### 6.3 Lack of intersectional frameworks

A further, often overlooked limitation is the absence of intersectional analysis in loneliness and cognitive aging research. While some studies consider sex or gender, few explore how these variables interact with race, ethnicity, socioeconomic status, disability, or sexual orientation—factors that profoundly shape both the experience and impact of loneliness (Gustafsson et al., 2022; Li and Spini, 2022).

For instance:

- Racially minoritized older adults may experience higher levels of structural isolation (e.g., residential segregation, language barriers), but lower reported loneliness due to cultural coping strategies (Batista-Malat et al., 2025; Taylor et al., 2024).
- Low-income women face elevated caregiving burdens and fewer formal support resources (Lalani et al., 2025), compounding both loneliness and dementia risk.
- LGBTQ+ older adults, particularly those without children or spousal supports, often navigate both social exclusion and under-recognition in aging research (Peterson et al., 2023; Pinazo-Hernandis et al., 2025).

The absence of intersectionality risks reinforcing a one-size-fits-all narrative, when in fact, the neurocognitive consequences of loneliness are shaped by the interplay of multiple identities (Gustafsson et al., 2022; Li and Spini, 2022). This limits the development of equitable public health strategies and obscures risk in underrepresented populations.

To advance aging neuroscience in a truly inclusive and mechanistically robust direction, the field must:

- Move beyond aggregated models by applying sex-disaggregated and intersectional analyses
- Expand how loneliness is conceptualized by incorporating rich, relational variables
- Recognize loneliness not just as a covariate, but as a central psychosocial determinant of cognitive health

Only with these enhancements can loneliness be fully understood and addressed as a multifactorial, gendered, and modifiable risk factor in the neuroscience of cognitive aging.

## 7 Toward a sex-sensitive framework for cognitive aging

As the evidence mounts connecting loneliness to cognitive decline, it becomes imperative to move beyond one-size-fits-all models of dementia risk and cognitive aging. The research reviewed in this paper points to loneliness as not only a modifiable factor but a sex-sensitive determinant that interacts with social, biological, and neurocognitive systems in distinct ways for women and men. This section proposes a conceptual model, highlights research gaps, and outlines actionable prevention strategies that are attuned to sex-specific patterns of vulnerability and resilience.

### 7.1 Conceptual model: loneliness as a modifiable, sex-sensitive risk factor

Emerging evidence supports a sex-sensitive conceptual model in which loneliness operates as a modifiable biopsychosocial exposure that interacts with gendered social roles, neuroendocrine stress responses, and immune-mediated brain vulnerability to shape cognitive aging trajectories. In this framework, loneliness is embedded within life-course exposures such as caregiving burden, widowhood, and reductions in emotionally supportive relationships—transitions that disproportionately affect women in later life and may compound cognitive risk through sustained psychosocial stress.

Evidence further suggests heightened susceptibility among women to these effects due to greater emotional investment in relationships, heightened social threat sensitivity, and neuroendocrine shifts—particularly in the postmenopausal period—that reduce resilience to social stress (Bekhbat and Neigh, 2018; Mengelkoch and Slavich, 2024; Ali et al., 2023). These sex-linked vulnerabilities are hypothesized to engage stress-responsive biological pathways, including hypothalamic–pituitary–adrenal axis dysregulation and chronic low-grade inflammation, which together may lower neural resilience in brain regions critical for memory, emotional regulation, and executive control.

Rather than reiterating individual findings, this framework synthesizes the reviewed literature to conceptualize loneliness—especially its emotional dimension—as a biologically embedded and socially patterned risk process. By integrating psychosocial context with neurobiological mechanisms, the model provides a foundation for sex-sensitive research designs and targeted prevention strategies aimed at mitigating loneliness-related cognitive vulnerability before clinical impairment emerges.

### 7.2 Implications for research: elevating sex-sensitive methodologies

Given the multidimensional and sex-specific pathways through which loneliness impacts cognitive aging, there is an urgent need for the field to adopt more rigorous, inclusive, and mechanistically-informed research practices. First, studies must routinely conduct sex-disaggregated analyses, rather than continuing the common practice of pooling data across sexes or merely adjusting for gender as a covariate. This includes testing for interaction effects between sex and loneliness, applying moderated mediation models, and stratifying neurocognitive outcomes to reveal potential divergences in both vulnerability and resilience mechanisms (Shen et al., 2022; Tao et al., 2022). Second, there is a clear need to improve the measurement of social and relational context, as current instruments often fail to capture critical dimensions such as emotional reciprocity, caregiving strain, and culturally gendered expectations. Expanded assessments should include indicators of relationship quality, caregiving demands, emotional versus social loneliness subtypes, and gendered life transitions like widowhood or retirement, which have been shown to shape loneliness experiences in sex-specific ways (Umberson et al., 2022; Htun et al., 2025). Lastly, to bridge psychosocial exposures and biological outcomes, future research must prioritize integrative longitudinal designs that combine self-reported loneliness and social data with biomarkers of stress and inflammation, neuroimaging findings, and long-term cognitive outcomes (Akhter-Khan et al., 2021; Warren, 2025). Only through this kind of interdisciplinary and sex-sensitive approach can we fully illuminate the mechanistic pathways linking loneliness to cognitive decline and identify early, modifiable targets for prevention.

### 7.3 Implications for prevention and intervention

Recognizing loneliness as a sex-sensitive and modifiable risk factor carries significant implications for clinical practice and public health intervention. First, targeted screening in primary care settings should become standard, particularly for older adults at elevated risk—such as women living alone, recent widows, and informal caregivers. These groups have consistently shown higher rates of emotional loneliness, which has distinct neurocognitive correlates compared to social isolation. Screening tools should therefore distinguish between emotional and social loneliness, as each subtype is associated with different patterns of brain aging and psychological distress (Chi et al., 2024; Tao et al., 2022).

Second, effective intervention strategies should be person-centered and adaptable, recognizing heterogeneity in preferences, needs, and responses both within and across sexes. Existing systematic reviews emphasize that loneliness interventions vary widely in format, mechanism, and effectiveness, with no single approach demonstrating universal benefit (Blodgett et al., 2025; Morrish et al., 2023; Veronese et al., 2021). Rather than supporting sex-specific prescriptions, this literature underscores the importance of matching intervention components to individual psychosocial profiles, contextual constraints, and readiness for engagement. Flexible delivery modalities (including digital and hybrid approaches such as telehealth, virtual companionship, or moderated online communities) may enhance accessibility and personalization, allowing interventions to be tailored without relying on gendered assumptions.

Finally, these insights support the case for precision prevention frameworks that integrate psychosocial data with biological and cognitive markers. By stratifying risk based on combined emotional, social, and neurobiological profiles, clinicians and public health systems can intervene before clinical symptoms emerge, enhancing the efficacy of dementia prevention efforts. Tailoring such strategies by sex and intersectional identity (e.g., socioeconomic status, race, and caregiving roles) can further improve health equity and resource targeting. Building a truly sex-sensitive framework for cognitive aging means centering loneliness not as a secondary symptom but as a biopsychosocial exposure with measurable and modifiable effects. With better tools, more inclusive models, and longitudinal data, this approach holds the promise of earlier detection, greater precision in risk mitigation, and more equitable cognitive health outcomes for aging women and men alike.

## 8 Conclusion

Loneliness is increasingly recognized not only as a widespread public health concern, but as a biologically consequential risk factor for cognitive decline. It is common in late life, affecting nearly one in three older adults globally, and yet remains under-prioritized in the domains of neuroscience, geriatrics, and public health. The evidence reviewed across epidemiological, neurobiological, and psychosocial studies makes clear that loneliness is more than a social state—it is a modifiable, sex-sensitive pathway to brain vulnerability.

Critically, the impact of loneliness is not uniformly distributed. Across nearly all dimensions reviewed (e.g., structural brain changes, functional connectivity, inflammatory response, and cognitive performance) women demonstrate greater vulnerability to the neurocognitive consequences of emotional loneliness. These effects are magnified by gendered life transitions (e.g., widowhood, caregiving), social roles, and hormonal changes, yet are rarely accounted for in conventional cognitive aging models.

This evidence presents a transformative opportunity for precision brain health. By explicitly incorporating loneliness into risk models, not as a peripheral covariate but as a central psychosocial exposure, researchers and clinicians can identify high-risk individuals earlier and intervene more effectively. Furthermore, adopting sex-sensitive analytic frameworks can improve the validity, equity, and translational power of both aging science and dementia prevention strategies.

The path forward requires interdisciplinary integration. Aging neuroscience must partner more closely with social epidemiology, gender studies, clinical psychiatry, and prevention science to develop tools, metrics, and interventions that reflect the real-world complexity of cognitive aging. Only through such cross-sector collaboration can we design systems of care and research that are responsive to the lived experiences of aging women and men alike, and that move the field toward early, equitable, and effective prevention of neurodegenerative disease.
